# A rare case of gastric cancer associated with gastric diverticulum

**DOI:** 10.1097/MD.0000000000046542

**Published:** 2025-12-19

**Authors:** Shaopeng Cheng, Weifeng Lan, Kezhong Tang, Congyan Zheng

**Affiliations:** a Department of Surgery, Suichang County People’s Hospital, Lishui, PR China; b Department of Surgery, Second Affiliated Hospital, Zhejiang University School of Medicine, Hangzhou, PR China; c Department of Nutrition, Suichang County People’s Hospital, Lishui, PR China.

**Keywords:** diverticulum, gastric cancer, surgery

## Abstract

**Rationale:**

Gastric cancer associated with prepyloric diverticulum is the most uncommon form of gastric cancer. It may be very challenging to diagnose as the lesion located among gastric antrum, pancreas, and transverse colon. We here reported a rare case of gastric cancer associated with gastric diverticulum involved part of transverse colon and head of pancreas.

**Patient concerns:**

Abdominal pain and vomiting for 5 days.

**Diagnoses:**

First diagnosis: colon cancer. Final diagnosis: gastric cancer from gastric diverticulum.

**Interventions:**

Three cycles of chemotherapy and operation.

**Outcomes:**

Regular follow-up 1 month after operation showed satisfactory recovery with no tumor recurrence.

**Lessons:**

This case underscores the importance of considering malignancy in atypical diverticular lesions.

## 1. Introduction

Gastric diverticulum (GD) can be divided into congenital and acquired. It is very rare and the rate of detection by endoscopy ranges from 0.01% to 0.11%.^[1]^ GD are usually asymptomatic. On occasion, the symptoms may include abdominal pain, dyspepsia, fatigue, nausea, and vomiting. GD was considered has little risk of malignant transformation, only 5 cases reported the possible association of GD with malignancy.^[2–6]^ In most cases, gastric cancer (GC) can present as a false GD to confuse the diagnosis. Here we reported a rare case of GC associated with GD in prepyloric region diagnosed based on final pathology after operation. The patient is a 58-year-old man with abdominal pain and vomiting for 5 days as his chief complaint. The patient was 1st diagnosed as colon cancer after biopsy and received 3 cycles of chemotherapy because of the confusion location of the lesion. After that he received exploratory laparotomy. During the operation, we found the tumor comes from GD, and the final pathology after operation also confirmed the diagnosis.

## 2. Case presentation

A 58-year-old man presented to the emergency department of his local hospital with a 5-days history of abdominal pain and vomiting. He denied hematemesis, melaena, weight loss, and changes in bowel habit. He was admitted to his local hospital because of mild hydrocephalus. He has suffered from gastric ulcer for some years. There are no other diseases, and the family history was unremarkable. There were no remarkable findings on physical examination.

## 3. Investigations

**Figure 1.**
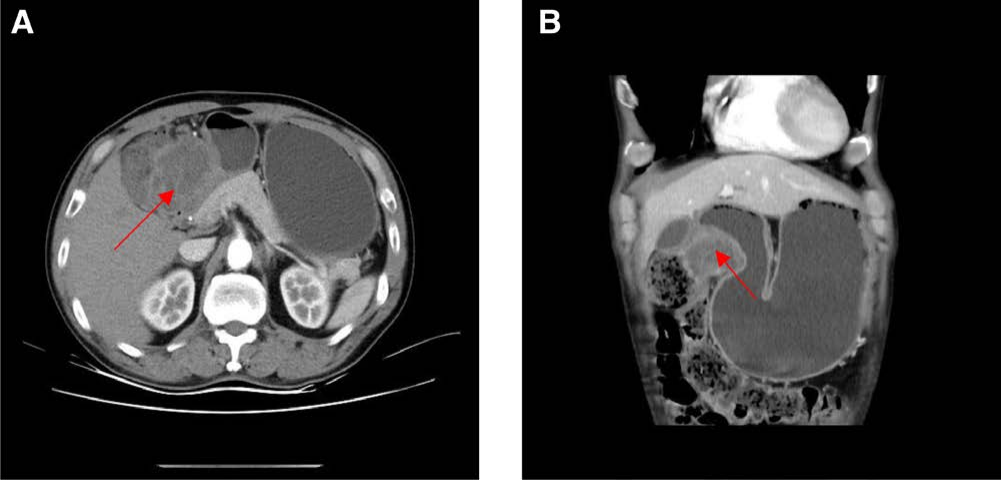
Contrast-enhanced CT scan on admission showed a mass measuring 5.2 cm in maximum diameter located among gastric antrum, pancreas, and transverse colon, and involved part of transverse colon and head of pancreas (arrow). (A) Transverse section; (B) coronal position.

Laboratory examinations on admission revealed the following: hemoglobin. 161 g/L (normal range: 130–175 g/L); leucocyte, 8.75 × 10^9^/L (normal range: 3.50–9.50 × 10^9^/L); C reactive protein, 1.5 mg/L (normal range: 0.0–10.0 mg/L); carcinoembryonic antigen, 5.57 ng/mL (normal range: <5 ng/mL); CA199, 48.98 U/mL (normal range: 0.00–34.00 U/mL). Contrast-enhanced CT scan on admission demonstrated a mass measuring 5.2 cm in maximum diameter located among gastric antrum, pancreas, and transverse colon, and involved part of transverse colon and head of pancreas (Fig. 1).

**Figure 2.**
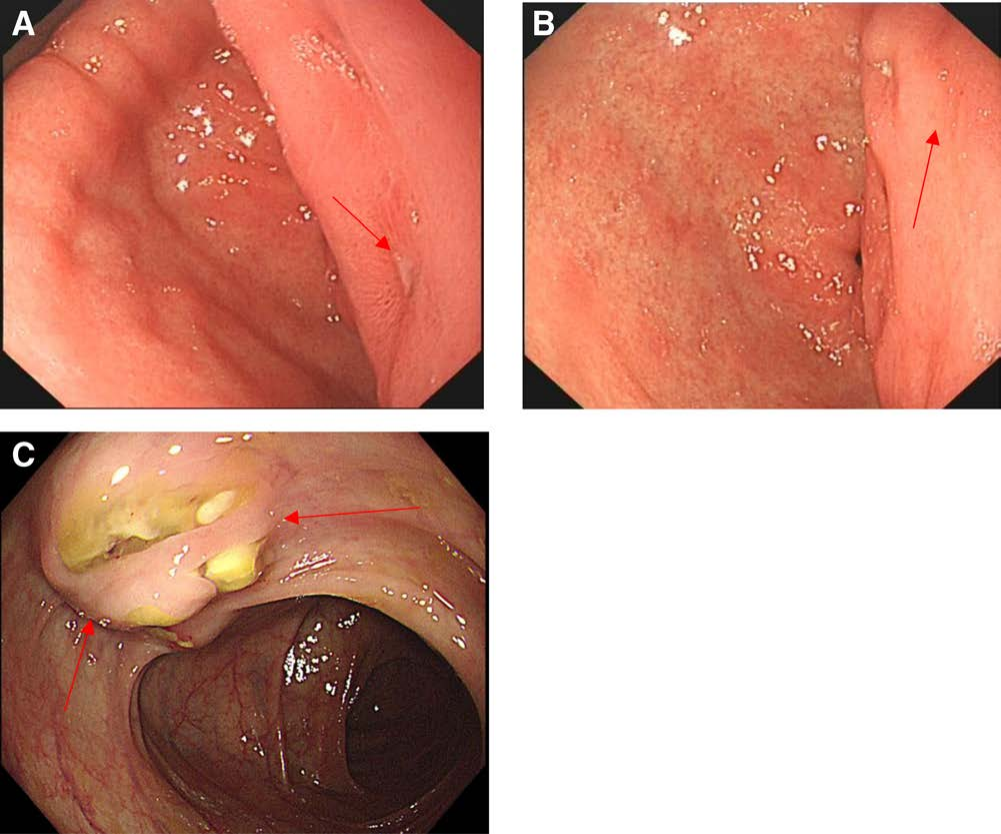
Results of gastroscopy and colonoscopy. (A, B) Images of gastroscopy; (C) image of colonoscopy. The patient was discovered to have prepyloric diverticulum near the gastric antrum (A arrow) and uplift due to external compression around the diverticulum (B arrow) during gastroscopy. A mass with rupture in transverse colon was found during colonoscopy (C arrow).

The patient was discovered to have prepyloric diverticulum near the gastric antrum and uplift due to external compression around the diverticulum during gastroscopy (Fig. 2A and B). A mass with rupture in transverse colon was found during colonoscopy (Fig. 2C). The following biopsy was performed during colonoscopy. The pathology of biopsy indicates poorly differentiated adenocarcinoma and signet ring cell carcinoma.

## 4. Treatment and outcome

**Figure 3.**
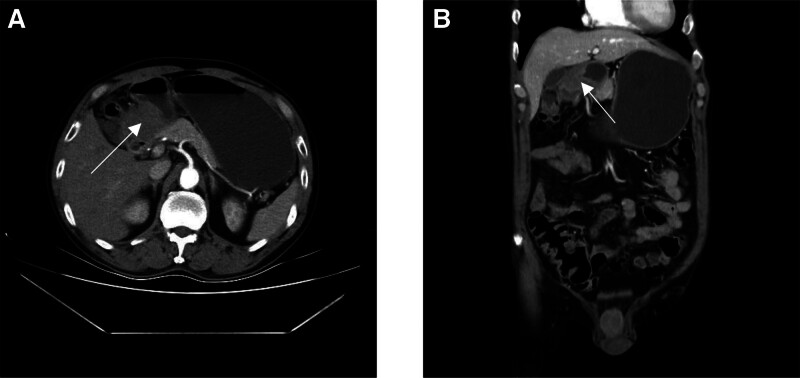
Contrast-enhanced CT scan after 3 cycles of neoadjuvant chemotherapy showed the diameter of the tumor reduced from 5.2 cm on admission to 4.2 cm (arrow). (A) Transverse section; (B) coronal position.

**Figure 4.**
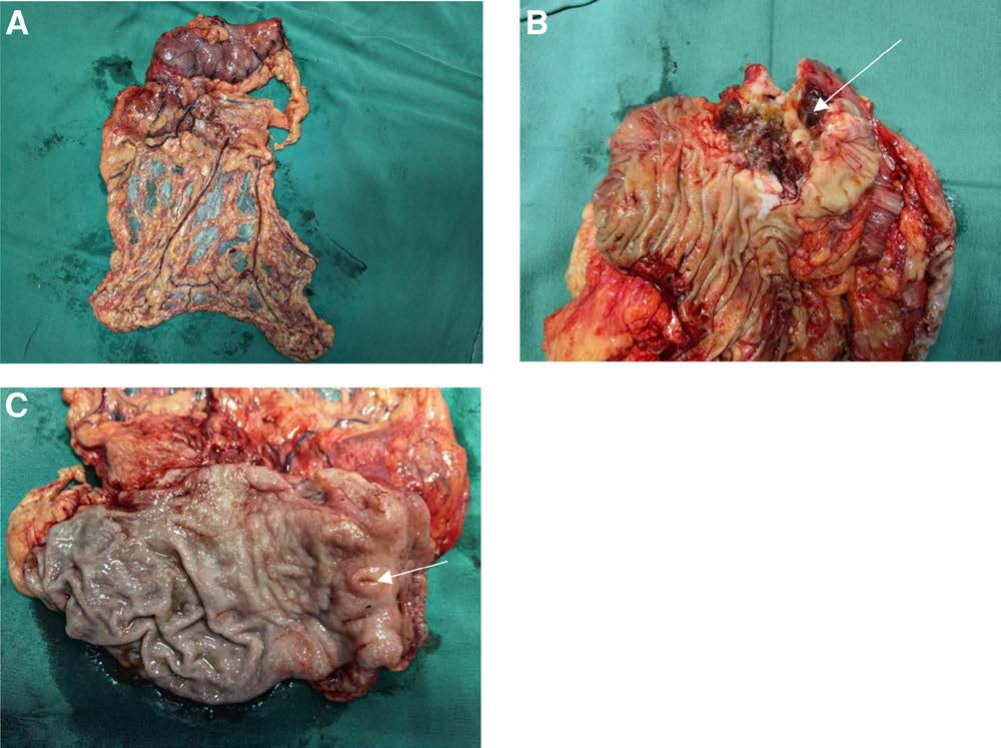
Resected tissue after operation. (A) Whole scope of resected tissue; (B) scope of tissue cut along transverse colon, tumor invaded part of colon (arrow); (C) scope of tissue cut along stomach. Gastric diverticula connect to tumor (arrow).

**Figure 5.**
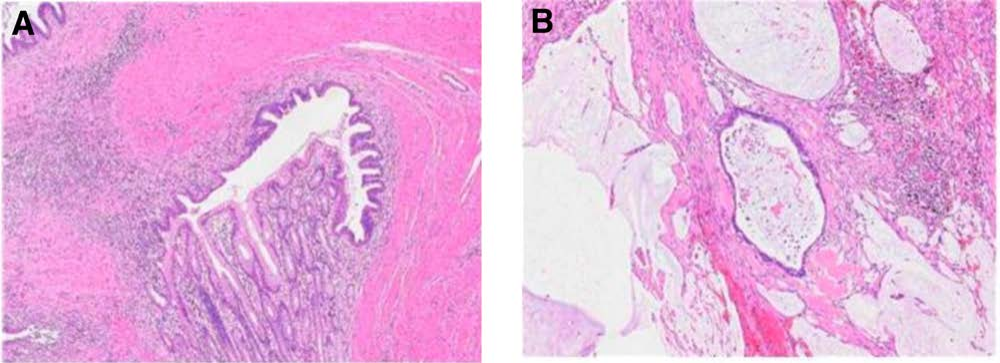
(A) Pathology of resected tissue after operation. (B) The final pathology report are mucinous adenocarcinoma from stomach, about 3.5 × 3 × 2 cm.

Based on the result of tissue biopsy and imaging examination, a recommendation was made to proceed 3 cycles of chemotherapy of capecitabine plus oxaliplatin with bevacizumab targeted therapy. reevaluation was performed after 3 cycles of neoadjuvant chemotherapy. Contrast-enhanced CT and MRI scan indicated the diameter of the tumor reduced from 5.2 cm on admission to 4.2 cm (Fig. 3). The level of CA199 changed from 48.98 U/mL on admission to 26.12 U/mL. The disease was partial regression according to criteria of tumor recist 1.1. The exploratory laparotomy was performed for the patient after multidisciplinary discussion. We found the lesion located among gastric antrum, pancreas, and transverse colon, still involved part of gastric antrum, transverse colon, and head of pancreas. So, we performed radical distal gastrectomy and partial transverse colectomy. For the tumor involved part of pancreatic tissue away from main pancreatic duct, and the location of the tumor is above duodenal papilla, we isolated tumor after resect part of pancreas and cut duodenum above duodenal papilla using Endo-Gia. From the resected tissue, we found the tumor has infiltrated the whole layer of transverse colon. There is a small hole with diameter of 0.5 cm in posterior wall of stomach, which is connected to the tumor. The posterior wall of stomach was partial involved by the tumor (Fig. 4). The final pathology after multidisciplinary discussion with the Second Affiliated Hospital of Zhejiang University confirmed the malignant tumor is from stomach (Fig. 5). The patient was allowed to eat food 3 days after the operation. The peritoneal cavity drainage tube was removed 5 days after the operation. The patient discharge 7 days after the operation with no complications, such as pancreatic fistula and anastomotic fistula. The results of regular follow-up 1 month after operation showed satisfactory recovery with no tumor recurrence.

## 5. Discussion

GD can be divided into congenital/true diverticula and acquired/false diverticula. Congenital diverticula were more common.^[7,8]^ Compared with false diverticula, true diverticula contain all layers of the gastric wall. According to the cause for false diverticula, false diverticula can be classified into 2 types, pulsion and traction. Pulsion diverticula can be caused by increased intraluminal pressure, such as chronic cough and obesity. While traction diverticula occur as a result of contractile forces, either from an adjacent inflammatory process, or from peri gastric adhesions from coexisting illnesses. Many diseases such as peptic ulcer, pancreatitis, cholecystitis, cancer, and gastroesophageal reflux can cause traction diverticula. The location of the 2 types of GD is also different. For congenital diverticula, they are mainly situated along the dorsal wall of the fundus, 2 to 3 cm from gastroesophageal junction and 3 cm from the lesser curve.^[8]^ However, acquired diverticula are typically situated near the gastric antrum.^[8]^

Up to now, only 5 cases reported the possible association of GD with malignancy.^[2–6]^ Fork FT et al^[3]^ reported an early GC in a fundic diverticulum, and the patient recovered after surgical removal of the gastric remnant. Oya M et al^[4]^ reported adenocarcinoma arising in a gastric partial diverticulum in the upper portion of the stomach presenting as a long-standing submucosal tumor. The patient recovered after laparoscopic wedge resection of the lesion. Lee YI et al^[5]^ performed endoscopic submucosal dissection of an inverted early gastric cancer-forming false GD located in the lesser curvature of the antrum. Olmez S et al^[6]^ just showed a rare case of coexistence of GD and gastric cancer. The malignant transformation process of gastric diverticula is complex and results from many factors, including inflammation, nutrition, and immune regulation. Recent evidence has shown that systemic inflammatory and nutritional indices, such as the platelet–albumin ratio and albumin-to-globulin ratio, are strongly associated with the development and prognosis of gastrointestinal malignancies.^[9,10]^ Moreover, integrative transcriptomic and single-cell analyses have identified immune regulators like IL27RA and TMEM71 as critical modulators of the tumor immune microenvironment and predictors of immunotherapeutic response.^[11,12]^ Among these, many molecular signaling pathways, such as cancer-associated fibroblasts related mechanism and cancer-associated adipocytes regulated pathways, play crucial roles in tumor development and progression.^[13,14]^

Differential diagnosis between GD and GC is made using a combination of upper gastrointestinal contrast radiographic studies, namely abdominal CT with oral contrast administration and use of endoscopy to visualize and obtain tissue from the suspected diverticulum. Orally administered contrast will typically opacify a GD, but not gastric cancer. Thickening of the wall of the cyst or signs of easy bleeding, superficial necroses, and a swollen fold arising from the tumor base during endoscopy test indicate possibility of malignant transformation. Endoscopic biopsy in high-risk region is needed. In this case, a mass located among gastric antrum, pancreas and transverse colon, and involved part of transverse colon and head of pancreas. Furthermore, there was no evidence of GD existing during endoscopy test, which confused us to make correct diagnosis at the first time. Abdominal CT with oral contrast administration and multiple-site biopsy with immunohistochemistry are the possible way to determine true origin of the tumor.

In this case, the patient has history of gastric ulcer for some years, which may be the cause of gastric cancer and diverticulum. One of the limitations of this case report is lack of imaging information before this hospitalization, which makes us hard to diagnose whether he has congenital diverticula. However, congenital diverticula in the prepyloric area are likely to be associated with aberrant pancreatic tissue,^[15]^ which is not consistent with findings in the case. According to the presentation of gastric tissue in Figure 4, the gastric mucosa is preserved well without infiltrated by gastric cancer. The possible diagnosis for this case is prepyloric gastric ulcer caused traction diverticula firstly, and then the ulcer and traction diverticula transformed to gastric cancer.

In most cases, gastric ulcer first transforms to cancer, and then cause gastric diverticula, which will cause gastric mucosa infiltration at the first time.^[16,17]^ While in the case, gastric mucosa preserved well during gastroscopy. Furthermore, we found a mass with rupture in transverse colon during colonoscopy. The pathology of biopsy indicates poorly differentiated adenocarcinoma. Depend on information obtained above, the 1st diagnosis of this case is transverse colon cancer after multidisciplinary discussion. This is a rare case of gastric ulcer causing traction diverticula, and then the ulcer and traction diverticula transforming to gastric cancer. The progression and location of the disease confused us to make correct diagnosis at the first time. However, the gastric cancer also showed encouraging responses to the neoadjuvant therapy based on 5-fluorouracil and oxaliplatin. In fact, multiple conventional chemotherapeutics agents, including 5-fluorouracil and oxaliplatin, also have the ability to mediate relevant immunostimulatory effects during cancer therapy.^[18,19]^

After 3 cycles of neoadjuvant chemotherapy, the disease was partial regression according to criteria of tumor recist 1.1. Considering the tumor was away from main pancreatic duct and partial invaded duodenal bulb, away from Vater ampulla, the tumor is resectable, with no need to perform Whipple, which could mostly preserve normal function of the body. So, we performed radical distal gastrectomy and partial transverse colectomy. For the tumor involved part of pancreatic tissue away from main pancreatic duct, and the location of the tumor is above duodenal papilla, we isolated tumor after resect part of pancreas and cut duodenum above duodenal papilla. The pathological margins of the tissue are all negative during intraoperative frozen section pathological examination. This is a complex case which is difficult to make correct diagnosis at the first time. Abdominal CT with oral contrast administration and multiple-site biopsy with immunohistochemistry should be performed at first time. The pathology result of the tissue resected found mucinous adenocarcinoma in gastric mucosa with part of pancreas and transverse colon invasion, which further confirmed the diagnosis of gastric cancer. Multidisciplinary collaboration is helpful during the whole treatment of the patient, including diagnosis, intervention and follow-up planning.

## 6. Conclusions

We reported a rare case of gastric cancer associated with prepyloric diverticulum involving part of transverse colon and head of pancreas. The distinguish progression and location of the lesion confused us to make correct diagnosis at the 1st time. The patient was 1st diagnosed as colon cancer and received 3 cycles of chemotherapy. During the operation, we found the tumor comes from GD, and the final pathology after operation also confirmed the diagnosis. Through this case, we found gastric diverticula also has the risk of a malignant transformation. The possible progression of the disease in this case is gastric ulcer causing traction diverticula 1st, and then the ulcer and traction diverticula transforming to gastric cancer.

## Author contributions

**Conceptualization:** Shaopeng Cheng, Congyan Zheng.

**Data curation:** Shaopeng Cheng.

**Investigation:** Shaopeng Cheng, Weifeng Lan.

**Methodology:** Weifeng Lan.

**Resources:** Weifeng Lan.

**Software:** Kezhong Tang.

**Supervision:** Kezhong Tang.

**Writing – original draft:** Shaopeng Cheng, Kezhong Tang, Congyan Zheng.

**Writing – review & editing:** Congyan Zheng.
